# Remote transient *Lactobacillus animalis* bacteremia causing prosthetic hip joint infection: a case report

**DOI:** 10.1186/s12879-016-1980-6

**Published:** 2016-11-04

**Authors:** R. Somayaji, T. Lynch, J. N. Powell, D. Gregson

**Affiliations:** 1 Departments of Medicine, University of Calgary, Calgary, AB Canada; 2 Pathology and Laboratory Medicine, University of Calgary, Calgary, AB Canada; 3 Surgery, University of Calgary, Calgary, AB Canada

**Keywords:** Bacteremia, Prosthetic joint infection, Lactobacillus

## Abstract

**Background:**

*Lactobacillus* spp. are uncommon pathogens in immunocompetent hosts, and even rarer causes of prosthetic device infections.

**Case presentation:**

A case of chronic hip prosthetic joint infection (PJI) caused by *L. animalis* is described. This occurred 5 years after a transient bacteremia with the same organism. Whole genome sequencing of both isolates proved this PJI infection resulted from this remote bacteremia.

**Conclusions:**

We document that prosthetic joint infections may be a consequence of bacteremia as much as 3 years before the onset of symptoms.

## Background

Bacteremic seeding of prosthetic joint infections is a concern for patients and care providers. Recent events are often scrutinized as a potential source of bactermia in such cases. This paper documents, using whole genome sequencing, that prosthetic joint infection with low virulence organisms may take years to present with local symptoms following an episode of bacteremia.

## Case presentation

A 70-year-old man was admitted to hospital in July of 2014 with progressively worsening left hip pain over 2 years with no associated neurologic symptoms. He had a medical history of Type 2 diabetes mellitus, remote history of a Whipple’s procedure for pancreatic cancer without recurrence, and bilateral total hip arthroplasties (THA) > 10 years previously. He had been admitted to hospital in October of 2009 for fever, nausea, and vomiting which he attributed to eating a turkey club sandwich. During this admission, he had blood cultures positive with *Lactobacillus* spp., for which he had received a 14-day course of clindamycin. A bone scan done to rule out a recurrence of his remote cancer showed mildly increased uptake in the hips bilaterally which was felt to be related to osteoarthritis and prior surgery. Attempts to aspirate the hips bilaterally failed to yield any fluid for analysis. His prosthetic joints functioned well for 3 years following this admission.

He subsequently developed gradually worsening left hip pain. He was therefore electively admitted to the orthopedic service for investigation. On examination in 2014, the patient was afebrile and hemodynamically stable. The neurologic exam did not reveal any abnormalities. Left hip mobility was decreased with pain, and the joint was painful to palpation. Pre-operative laboratory investigations revealed a hemoglobin level of 126 g/L, platelet count of 224 * 10^9^/L, white cell count of 10.4 * 10^9^/L with a neutrophil count of 7.6 * 10^9^/L, an ESR of 11 mm/h, C-reactive protein of 2.8 mg/L, and a creatinine of 43 μmol/L. Radiographs of the left hip revealed severe loosening of the arthroplasty and extensive peri-prosthetic lucency with significant progression since 2012. Two sets of blood cultures were drawn, and these were negative.

The patient was taken to the operating room and had the left hip implant removed with the placement of an antibiotic-embedded spacer. During surgery, purulent material in the left hip joint was noted. One of four intra-operative samples of fluid and tissue demonstrated Gram-positive bacilli on the Gram strain and three of the four cultures subsequently grew a *Lactobacillus* spp.*.* identified in the microbiology laboratory using mass spectrometry (Vitek MS®, Biomerieux Canada). Hip fluid was not sent for biochemical and cellular parameter due to the clinical picture during the operative procedure. The patient was managed using a 2 stage protocol with 6 weeks of IV antibiotics followed by 6 weeks of oral antibiotics active against the Lactobacillus isolated. Operative cultures in February of 2015 when a new prosthetic joint was inserted were negative.

Lactobacillus spp. are rarely reported as causes of prosthetic joint infections [1–3] and the remote bacteremia appeared to be a likely cause of this patient’s infection. To prove this, whole genome sequencing was performed. Briefly, the frozen isolate from 5 years previously (09-2317) and that from the current infection (14-7927) were sequenced using NexteraXT DNA sample preparation on a MiSeq instrument (Illumina CA USA) to generate paired- end 250 base pair sequences. The quality of data was assessed using FastQC (http://www.bioinformatics.babraham.ac.uk/projects/fastqc/). Read error correction was performed using BayesHammer in the de novo assembly process using SPAdes version 3.1.1 [4]. The quality of assemblies was assessed using Quast [5] and comparison of total genome content using GView Server [6].

**Fig. 1:**
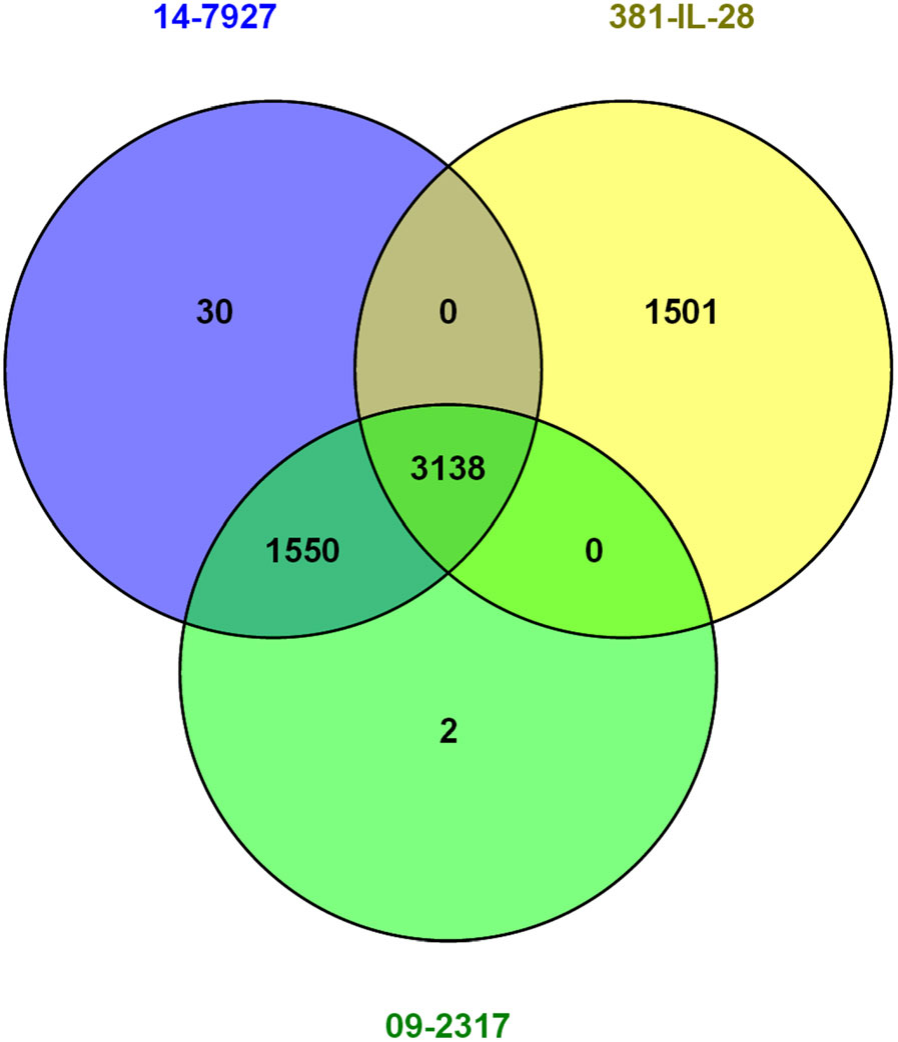
Venn diagram comparing core single nucleotide polymorphism (SNP) positions of *L. animalis* genomes: the two clinical isolates (09-2317, 14-7927) and a publicly available genome of the same species (381-IL-28) determined through alignment to the reference *L. animalis* genome (KCTC-3501)

Whole genome assemblies were compared using Parsnp to align both clinical isolates with two publically available *Lactobacillus animalis* genomes, KCTC 3501 and 381-IL-28 (Genbank accessions: AEOF01, JMHU01 respectively). Single nucleotide polymorphisms (SNPs) within the core genome were calculated using KCTC 3501 as the reference strain in Parsnp. SNP positions were compared using a Venn diagram generated with Venny [7] (Fig. 1).

**Fig. 2:**
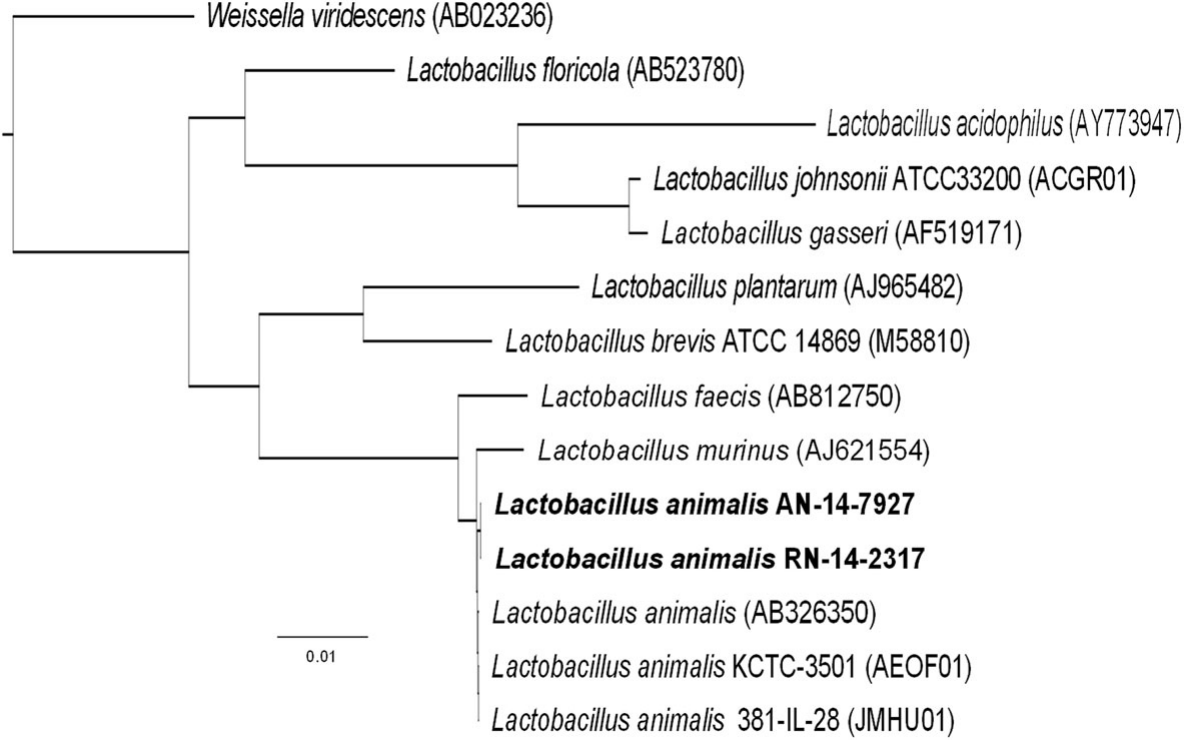
Similarity tree of clinical isolates ((09-2317,14-7927) using 16S rDNA sequencing and comparison to known 12 known publically available sequences. Available at https://treebase.org/treebase-web/search/studySearch.html. Search study S19258

Fourteen partial 16S rDNA sequences were analyzed, including two extracted gene sequences from the 14-7927 and 09-2317 assemblies and 12 publically available sequences [8]. Multiple sequence alignment was performed with MUSCLE version 3.8.31 [9] then inspected and trimmed with AliView version 1.15 [10] resulting in a 1492 bp alignment. A maximum likelihood tree was inferred with FastTree version 2.1.4 with a distribution of 1000 resampled trees under the generalized time reversible (GTR) model of evolution and the Shimodaira-Hasegawa test to calculate local support values [11] (Fig. 2).

As shown in Fig. 1, the lactobacilli isolated from this patient 5 years apart differed only by 32 core SNPs collectively with 4688 SNPs in common (99.32 % similarity) while the publically available 381-IL-28 genome had 1501 unique SNPs and 3138 in common with isolates from this patient (47.83 % similarity). It is highly unlikely that two unrelated isolates separated in time and location would be this closely related. This supports the conclusion of remote seeding to the prosthesis with a long asymptomatic period. Figure 2 identifies both of the isolates as *L. animalis.*

Bacteremia with *Lactobacillus* spp.. is uncommon. There are no prior reports of *L. animalis* bacteremia or infected joints. However, many isolates in prior publications were identified only to the genus level [12, 13] of which some were likely *L. animalis.* Internal laboratory review found only 8/3800 cases of bacteremia in our region in 2014. In the largest case series of 89 cases reported by Salminen et al [12], there was no documented seeding of prosthetic joints. Our case, therefore, represents an uncommon complication of an uncommon cause of bacteremia.

Bennett et al [14] have described a case of chronic prosthetic joint infection due to *Lactobacillus* spp*.*. In their case, no antecedent bacteremia was documented. They highlight concerns of probiotic foods being a potential source of such infections. However, even in cancer patients, organisms present in “probiotic” supplements rarely cause significant infections [15]. *L. rhamnosus* and *L. acidophilus* are the most common *Lactobacilli* spp*.* used in probiotics. *L. animalis* is not commonly listed as an active ingredient in probiotics or fermented foods, and this case seems to have occurred in the setting of food poisoning. Speciation and molecular typing of *Lactobacilli* spp. infections are, therefore, an important part of the investigations to determine the relationship between non-pasteurized fermented food products or live probiotic formulations.

## Conclusions

In summary, we present the first human case of *L. animalis* bacteremia and prosthetic joint infection following a documented episode of remote bacteremia. Whole genome sequencing, in this case, documents years between hematogenous seeding and clinical manifestations of infections of prosthetic joints with low virulence organisms. Attempts to attribute late prosthetic joint infections to specific events may be impossible to determine unless there is a documented bacteremia as occurred in this case.

## Abbreviations

spp: Species

## References

[1] Atwal N, George A, Squires B, Marsh CH (2009). Lactobacillus as a rare cause of an infected total knee replacement: a case report. J Med Case Rep.

[2] Orkaby AR, Chen B, Iliaki EF, Sulis CA, Oates DJ (2012). A curious case of Lactobacillus casei in a prosthetic joint: was it the yogurt?. J Am Geriatr Soc.

[3] Bereza PL, Ekiel A, Auguściak-Duma A, Aptekorz M, Wilk I, Kusz DJ (2013). Identification of silent prosthetic joint infection: preliminary report of a prospective controlled study. Int Orthop.

[4] Bankevich A, Nurk S, Antipov D, Gurevich AA, Dvorkin M, Kulikov AS (2012). SPAdes: a new genome assembly algorithm and its applications to single-cell sequencing. J Comput Biol.

[5] Gurevich A, Saveliev V, Vyahhi N, Tesler G (2013). QUAST: quality assessment tool for genome assemblies. Bioinformatics.

[6] Petkau A, Stuart-Edwards M, Stothard P, Van Domselaar G (2010). Interactive microbial genome visualization with GView. Bioinformatics.

[7] Oliveros JC. (2007-2015) Venny. An interactive tool for comparing lists with Venn's diagrams. http://bioinfogp.cnb.csic.es/tools/venny/. Accessed 8 Sept 2015.

[8] Quast C, Pruesse E, Yilmaz P, Gerken J, Schweer T, Yarza P (2013). The SILVA ribosomal RNA gene database project: improved data processing and web-based tools. Nucleic Acids Res.

[9] Edgar RC (2004). MUSCLE: multiple sequence alignment with high accuracy and high throughput. Nucleic Acids Res.

[10] Larsson A (2014). AliView: a fast and lightweight alignment viewer and editor for large datasets. Bioinformatics.

[11] Price MN, Dehal PS, Arkin AP (2010). FastTree 2--approximately maximum-likelihood trees for large alignments. PLoS One.

[12] Salminen MK, Rautelin H, Tynkkynen S, Poussa T, Saxelin M, Valtonen V (2004). Lactobacillus bacteremia, clinical significance, and patient outcome, with special focus on probiotic L. rhamnosus GG. Clin Infect Dis.

[13] Cannon JP, Lee TA, Bolanos JT, Danziger LH (2005). Pathogenic relevance of Lactobacillus: a retrospective review of over 200 cases. Eur J Clin Microbiol Infect Dis.

[14] Bennett DM, Shekhel T, Radelet M, Miller MD (2014). Isolated Lactobacillus chronic prosthetic knee infection. Orthopedics.

[15] Redman MG, Ward EJ, Phillips RS (2014). The efficacy and safety of probiotics in people with cancer: a systematic review. Ann Oncol.

